# Sleep disturbance among Malaysian patients with end-stage renal disease with pruritus

**DOI:** 10.1186/s12882-019-1294-1

**Published:** 2019-03-25

**Authors:** Inayat Ur Rehman, Pauline Siew Mei Lai, Soo Kun Lim, Learn Han Lee, Tahir Mehmood Khan

**Affiliations:** 1 School of Pharmacy, Monash University, Jalan Lagoon Selatan, Bandar Sunway, 47500 Selangor Darul Ehsan Malaysia; 2 Department of Pharmacy, Abdul Wali Khan University Mardan, Mardan, Pakistan; 3 Department of Primary Care Medicine, Faculty of Medicine, University of Malaya, Kuala Lumpur, Malaysia; 4 Department of Medicine (Division of Nephrology), Faculty of Medicine, University of Malaya, Kuala Lumpur, Malaysia; 5 Novel Bacteria and Drug Discovery Research Group, Microbiome and Bioresource Research Strength, Jeffrey Cheah School of Medicine and Health Sciences, Monash University Malaysia, 47500 Bandar Sunway, Selangor Darul Ehsan Malaysia; 6 Institute of Biomedical and Pharmaceutical Sciences, Guangdong University of Technology, 510006 Guangzhou, People’s Republic of China; 7 Center of Health Outcomes Research and Therapeutic Safety (Cohorts), School of Pharmaceutical Sciences, University of Phayao, Phayao, Thailand; 8 Institute of Pharmaceutical Sciences, University of Veterinary and Animal Science, Outfall Campus, Civil Lines, Lahore, Pakistan

**Keywords:** Chronic kidney disease associated pruritus, 5D itch scale, Pruritus, Sleep quality, Malaysia

## Abstract

**Background:**

Chronic kidney disease-associated pruritus (CKD-aP) is a well-recognized, frequent and compromising complication among patients on hemodialysis. Despite advancement in basic medical sciences, CKD-aP is still a major complication and a challenge for both physicians and patients to manage. The aim of this study was to estimate the prevalence of CKD-aP among hemodialysis patients in Malaysia, to determine the impact of CKD-aP on sleep quality and any factors associated with CKD-aP.

**Method:**

A multi-centered, cross-sectional study design was conducted from February 2017 to September 2017 at a tertiary hospital and its affiliated dialysis centers, in Kuala Lumpur, Malaysia. Included were patients > 18 years of age who were undergoing hemodialysis and could understand Malay. Participants were asked to fill the Malay 5D-itch scale and the Malay Pittsburgh sleep quality index (PSQI) upon recruitment.

**Results:**

A total of 334/334 patients were recruited (response rate = 100%). The majority were male (59.6%) and Chinese (61.7%). A total of 61.3% had pruritus, of which most patients (63.4%) reported that their pruritus was mild. More than half (54.1%) reported that they slept > 6 h, and 93.2% experienced no sleep disturbances during the night. However; the overall PSQI median score [IQR] was 6.0 [5.0–9.0]. No significant association was found between demographic and clinical characteristics of patients with the severity of pruritus. Patients with moderate to severe pruritus were found to be 5.47 times more likely to experience poor sleep quality as compared to patients with mild or no pruritus.

**Conclusion:**

In Malaysia, the prevalence of CKD-aP was 61.3%, of which the majority reported that their pruritus was mild. Patients with moderate to severe pruritus were found to be 5.47 times more likely to experience poor sleep quality as compared to patients with mild or no pruritus.

## Background

Chronic kidney disease associated-pruritus (CKD-aP) is a well-recognized, frequent and compromising complication among patients on hemodialysis. Treating CKD-P is a huge challenge [1, 2]. Treatment is difficult due to the refractory nature of the disease [3]. The absence of suitable and effective treatments for CKD-aP management has resulted in poor sleep among hemodialysis patients [3]. The prevalence of CKD-aP varies from country to country. This may be due to advancement in basic health and medical sciences in developed countries versus developing countries [4]. The prevalence of CKD-aP has decreased from 90% [5] in the 1970s to 22–55% in the modern era [6–8].

The pathophysiology of CKD-aP is not completely clear [1] and is believed to be multifactorial, resulting from the integration of multiple factors’ including demographics, neuropathic and psychogenic factors [9]. During hemodialysis, numerous cytokines including interleukin-1 are released following contact with plasma and the dialysis membranes [10]. Interleukin-1 has been suggested to stimulate the release of possible pruritogenic substances [11]. It is also suggested that in hemodialysis patients, accumulation of non-dialyzable middle molecules stimulate free nerve endings contributing to pruritus [11].

CKD-aP usually begins about six months after the start of dialysis [12], and studies have shown a significant positive relationship between pruritus and the duration of hemodialysis [13]. Pruritus can present as either an acute or chronic condition [7], either generalized or localized; and may last from a few months to in some cases more than one year [14]. The onset of pruritus, its duration, and its intensity can change over time, and itch has been reported to worsen at night [15]. The body parts affected are the back, limbs, chest, and head [15].

Pruritus is an undesirable situation that aggravates itchiness and negatively affects patients’ sleep as well as the quality of life [16, 17] as it causes nocturnal awakenings and creates difficulty in falling asleep [18, 19]. A higher mortality risk is also observed in those with poor sleep quality [20]. Moderate to extreme CKD-aP had 15–21% higher mortality compared to those without pruritus [15]. Therefore, the aim of this study was to estimate the prevalence of CKD-aP among hemodialysis patients in Malaysia, to determine the impact of CKD-aP on sleep quality and any factors associated with CKD-aP.

## Methods

A cross-sectional, multi-centered study was conducted from February to September 2017 at a tertiary hospital and its affiliated dialysis centers in Kuala Lumpur, Malaysia.

### Participants

Patients ≥18 years of age who were undergoing hemodialysis and could understand Malay were recruited. Excluded were patients with pruritus due to other conditions such as liver disease, *systemic lupus erythematosus (*SLE).

### Sample size

Sample size was calculated using the Cochran formula [21]. Our pilot study found that the prevalence of CKD-aP was 68% [22]. Hence, the sample size required was 334 (with a 95% level of confidence and 80% power).

### Instruments used

#### Baseline demographic questionnaire

This questionnaire was used to collect demographic data such as socioeconomic status, disease duration, dialysis duration, and existing comorbidities conditions.

#### The Malay 5D-itch scale (MD-IS)

The validated Malay 5D-itch scale (MD-IS) [22] was used to assess CKD-aP. The Malay 5D-itch scale has five domains, addressing the duration, degree, direction, disability, and distribution of itching. All items of the four domains (duration, degree, direction, disability) were measured on a five-point Likert scale (where 1 represented the lowest degree of pruritus, and 5 represented the highest degree). The distribution domain was measured in a different manner. If two body parts were affected, the score given was 1; 3–5 body parts affected was scored as 2, 6–10 body parts were scored as 3, 11–13 body parts were scored as 4, and 14–16 body parts were scored as 5. The overall score of the MD-IS was calculated by summing all five domains; a score below 5 indicates no pruritus; whilst a score of 25 indicates severe pruritus [22]. M5D-IS score of 5–10 indicates mild pruritus, a score of 11–19 indicated moderate pruritus and 20–25 shows severe pruritus.

#### The Malay Pittsburgh sleep quality index (PSQI)

The validated Malay Pittsburgh sleep quality index (PSQI) [23] is an instrument that measures self-rated sleep quality over the past one month. It consists of 19 items, which is combined with seven component scores such as subjective sleep quality, sleep latency, sleep duration, habitual sleep efficiency, sleep disturbances, use of sleep medication, and daytime dysfunction. Sleep duration was rated on a 4-point Likert scale of less than 5 h to more than 7 h; sleep efficiency was rated by the number of hours asleep divided by the total number of hours in bed. The use of sleep medication and poor daytime dysfunction were rated on a 4-point Likert scale (“not during the past month, less than once per week, once or twice per week, three or more times per week”). Sleep disturbance was assessed by nine questions which focused on waking up in the middle of the night or early in the morning, getting up to go to the toilet, difficulty breathing properly, coughing or snoring loudly, being too cold, being too hot, having nightmares, experiencing pain, or other reasons for disturbed sleep and each question was rated on 4-point Likert scale (“not during the past month, less than once per week, once or twice per week, three or more times per week”). Subjective sleep was evaluated with one question on a 4-point Likert scale from very good to very bad. The seven components were each scored from 0 (no difficulty) to 3 (severe difficulty) [24, 25]. The overall score was combined by summing up the scores of these seven components that ranged from 0 to 21 [26]. PSQI score ≥ 5 were classified as bad sleepers and PSQI < 5 classified were as good sleepers [26].

### Procedure

Patients were approached while they were undergoing hemodialysis. The objective of the study was explained. Written informed consent was obtained from those who agreed to participate. Participants were then asked to fill in the baseline demographic form, the Malay 5D-itch scale and the Pittsburgh sleep quality index (PSQI).

### Statistical analysis

Data were analyzed using the Statistical Package for Social Sciences version 20.0 (SPSS Inc., Chicago, IL). Normality was assessed using the Kolmogorov–Smirnov test. As normality could not be assumed, non-parametric tests were used. Categorical variables were presented as number and frequency, while continuous variables were presented as either mean or standard deviation or median and interquartile ranges (IQR). To determine the factors associated with pruritus, patients were categorized to 1) mild pruritus and 2) moderate to severe pruritus. Any association between variables which had a *p*-value < 0.25 were then included in the logistic regression analysis, where a *p*-value< 0.05 was considered significant.

## Results

**Fig. 1:**
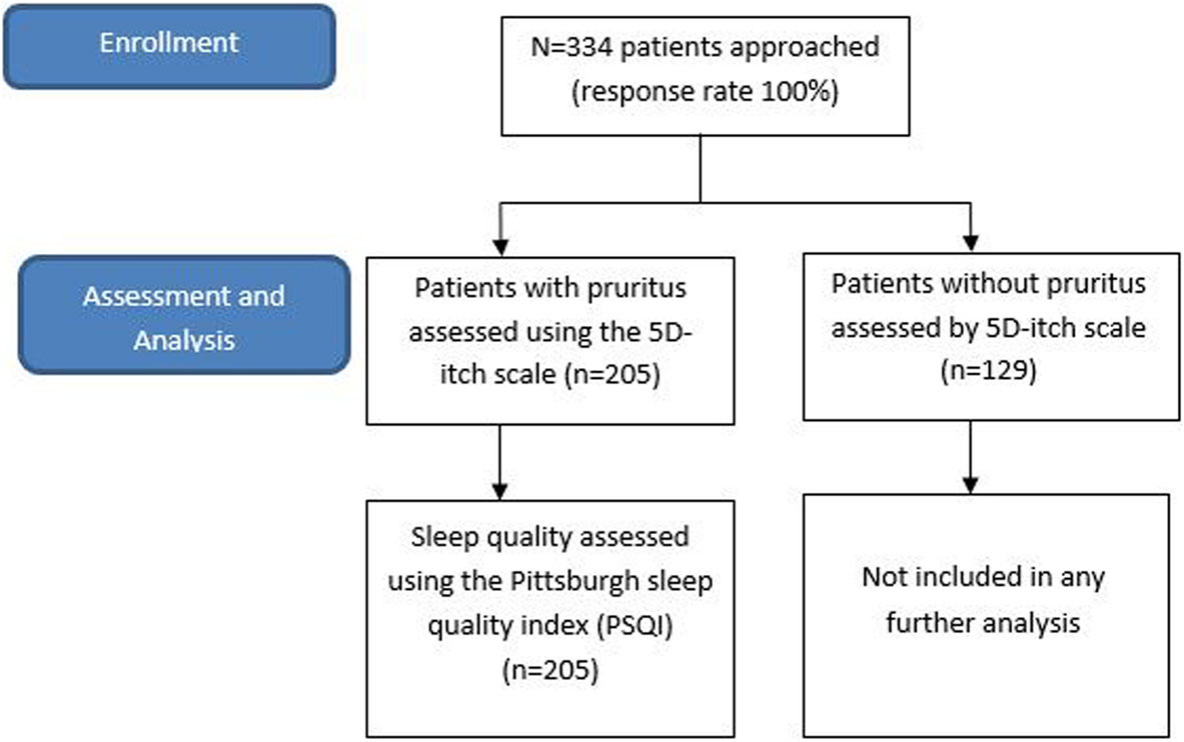
Flow diagram of patient recruitment and eligibility for further assessment

A total of 334/334 patients were recruited (response rate = 100%). The flow on how patients were recruited is shown in Fig. 1. The majority were male (59.6%) and Chinese (61.7%). Significantly more Malays had pruritus (*p* = 0.014). The duration of having CKD and duration in months receiving dialysis were significantly higher in patients with pruritus. Hypertension (*n* = 142), diabetes mellitus (*n* = 85), and hyperlipidemia (*n* = 30) were the most common co-morbidities observed in CKD patients with pruritus (Table 1).

**Table 1:** Demographics and clinical characteristic of Participants (*n* = 334)

Statement	N	No pruritus *N* = 129 N (%)	Pruritus *N* = 205 N (%)	*P*-value
Gender				
*Male*	199	81 (40.7)	118 (59.3)	0.343 ^**a**^
*Female*	135	48 (35.6)	87 (64.4)	
Ethnicity				
*Malay*	70	19 (27.1)	51 (72.9)	0.014* ^**a**^
*Chinese*	206	80 (38.8)	126 (61.2)	
*Indian*	58	30 (51.7)	28 (48.2)	
Age				
Median age in years [IQR]		58.00 [47.00–67.00]	59.00 [49.00–68.00]	0.558 ^**c**^
*18–30 years*	9	4 (44.4)	5 (55.6)	0.961 ^**a**^
*31–40 years*	28	11 (39.3)	17 (60.7)	
*41–50 years*	66	28 (42.4)	38 (57.6)	
*51–60 years*	85	32 (37.7)	53 (62.3)	
*61–70 years*	88	31 (35.2)	57 (64.8)	
*70 years and above*	58	23 (39.7)	35 (60.3)	
Median duration of having CKD in months [IQR]		36.00 [24.00–84.00]	60.00 [24.00–132.00]	0.002* ^**c**^
*< 1 year*	26	15 (57.7)	11 (42.3)	0.044* ^**a**^
*1–2 years*	80	38 (47.5)	42 (52.5)	
*3–4 years*	67	28 (41.8)	39 (58.2)	
*5–6 years*	42	12 (28.6)	30 (71.4)	
*7–8 years*	33	12 (36.4)	21 (63.6)	
*9–10 years*	22	6 (27.3)	16 (72.7)	
*11 years and more*	64	18 (28.1)	46 (71.9)	
Median duration in months receiving dialysis [IQR]		36.00 [12.00–60.00]	48.00 [24.00–91.00]	0.004* ^**c**^
*< 1 year*	33	21 (63.6)	12 (36.4)	0.009* ^**a**^
*1–2 years*	89	40 (44.9)	49 (55.1)	
*3–4 years*	77	28 (36.4)	49 (63.6)	
*5–6 years*	42	13 (31.0)	29 (69.0)	
*7–8 years*	33	11 (33.3)	22 (66.7)	
*9 years and above*	60	16 (26.7)	44 (73.3)	
Frequency of dialysis #				
*Twice a week*	2	0 (0)	2 (100)	0.148 ^**b**^
*Three times a week*	332	129 (38.9)	203 (61.1)	
Presence of co-morbidities**				
Diabetes mellitus	110	25 (22.8)	85 (77.2)	
Hypertension	170	28 (16.5)	142 (83.5)	
Hyperlipidemia	40	10 (25)	30 (75)	
Cardiovascular diseases	6	NA	6 (100)	
Gout	7	NA	7 (100)	
Goiter	5	NA	5 (100)	

#High flux dialyzer were used, (membranes used in these dialyzers were polyarylsulfone family (Polysulfone, polyarylsulfone, Polyarylethersulfone and polyethersulfone membrane) Polyester polymer alloy (PEPA) *membrane; which have* two polymers, polyethersulfone and polyarylate)

; a: Chi-square test; b: Fisher exact test; c: Mann-Whitney U test; *p < 0.05; *NA* not available

**(Diabetes mellitus, hypertension, hyperlipidemia) were the most common comorbidities observed in current cohort of patients. Figures were > 100% as patients may be suffering from more than one chronic condition

### Prevalence and the characteristic of CKD-aP

Of the 334 patients recruited, 205 (61.3%) reported that they had CKD-aP based on the total score of the M5D-itch scale (Table 2). Most patients *n* = 182 (88.1%) reported that they experienced pruritus for < 6 h a day and 130 (63.4%) patients reported that their intensity of itching over the past 2 weeks was mild, the remaining participants reported that the intensity of itching was moderate [16 (7.8%)] and severe [6 (2.9%)] (Table 2). The majority [133 (64.9%)] reported that their pruritus has completely resolved. Nearly half [100 (48.8%)] quoted pruritus as a reason for the difficulty in falling asleep. The majority of participants (> 77.0%) also reported that pruritus did not affect their leisure, housework or work/school. Back, forearm, abdomen, lower leg, and thighs, were parts of the body most affected by pruritus. Most patients [177 (86.3%)] had mild pruritus (based on the overall total score of M5D itch scale score of 5–25) as shown in Table 2. All patients (205%?) were dialyzed using high-flux dialyzers. The membranes used in these dialyzers were of the polyarylsulfone family. Seven (3.4%) patients were prescribed antihistamines, 4 (1.9%) were prescribed steroids, and 2 (0.9%) were prescribed gabapentinoids.

**Table 2:** Responses obtained by patients regarding CKD-aP using the Malay 5D itch scale s (*n* = 205)

Domain	Statement	Responses	N (%)
Duration	During the last 2 weeks, how many hours a day have you been itching?	Median [IQR]	1.0 [1.0–1.0]
		Less than 6 h/day	182 (88.1)
		6–12 h/day	17 (8.3)
		12–18 h/day	1 (0.5)
		18–23 h/day	1 (0.5)
		All day	4 (2.0)
Degree	Please rate the intensity of your itching over the past 2 weeks	Median [IQR]	2.0 [1.0–2.0]
		Not present	53 (25.9)
		Mild	130 (63.4)
		Moderate	16 (7.8)
		Severe	6 (2.9)
		Unbearable	0
Direction	Over the past 2 weeks has your itching gotten better or worse compared to the previous month?	Median [IQR]	1.0 [1.0–2.0]
		Completely resolved	133 (64.9)
		Much better but still present	61 (29.8)
		Little bit better but still present	7 (3.4)
		Unchanged	3 (1.5)
		Getting worse	1 (0.5)
Disability: Sleep	Rate the impact of your itching on the following activities over the last 2 weeks	Median [IQR]	2.0 [1.0–2.0]
		Never affects sleep	78 (38.0)
		Occasionally delays falling asleep	100 (48.8)
		Frequently delays falling asleep	20 (9.8)
		Delays falling asleep and occasionally wakes me up at night	5 (2.4)
		Delays falling asleep and frequently wakes me up at night	2 (1.0)
Disability: Leisure/Social	Rate the impact of your itching on the following activities over the last 2 weeks	Median [IQR]	1.0 [1.0–1.0]
		Never affect activity	159 (77.6)
		Rarely affects activity	24 (11.7)
		Occasionally affects activity	9 (4.4)
		Frequently affects activity	6 (2.9)
		Always affects activity	7 (3.4)
Disability: Housework/Errands	Rate the impact of your itching on the following activities over the last 2 weeks	Median [IQR]	1.0 [1.0–1.0]
		Never affect activity	161 (78.5)
		Rarely affects activity	22 (10.7)
		Occasionally affects activity	11 (5.4)
		Frequently affects activity	7 (3.4)
		Always affects activity	4 (2.0)
Disability: Work/School	Rate the impact of your itching on the following activities over the last 2 weeks	Median [IQR]	1.0 [1.0–1.0]
		Never affect activity	178 (86.8)
		Rarely affects activity	14 (6.8)
		Occasionally affects activity	6 (2.9)
		Frequently affects activity	3 (1.5)
		Always affects activity	4 (2.0)
Distribution bin score	Mark whether itching has been present in the following parts of your body over the last 2 weeks. If a body part is not listed, choose the one that is closest anatomically.	Median [IQR]	1.0 [1.0–1.0]
		Score bin 1	106 (51.7)
		Score bin 2	85 (41.5)
		Score bin 3	10 (4.9)
		Score bin 4	1 (0.5)
		Score bin 5	3 (1.5)
Total no. of participants with pruritus			205 (100.0)
Total score of 5D itch scale	Ranged from 5 to 25	Median [IQR]	8.0 [6.0–9.0]
		5–10 (Mild pruritus)	177 (86.3)
		11–19 (Moderate pruritus)	26 (12.7)
		20–25 (severe pruritus)	2 (1.0)
Type of dialyzers used		High flux dialyzers	205 (100)

*IQR* interquartile range

More than half (54.1%) of patients reported that they slept > 6 h, 191 (93.2%) experienced no sleep disturbances during the night. Majority of the patients [107 (52.2%)] of the patients were having mild difficulty score in sleep latency, whereas 144 (70.2%) had mild difficulty in day time dysfunction, 85 (41.5%) reported that their sleep efficiency was 85% and 141 (68.8%) reported that their sleep quality ranged from fairly to very good, 202 (98.5%) of our patients never used sleep medications. The overall PSQI median score [IQR] was 6.0 [5.0–9.0] (Table 3).

**Table 3:** Responses obtained by patients regarding their sleep quality using the Malay Pittsburgh Sleep Quality Index (PSQI) (*n* = 205)

Components of PSQI	Responses	Frequency	Percentage
Sleep duration	*> 7 h*	24	11.7
	*6–7 h*	88	42.9
	*5–6 h*	47	22.9
	*< 5 h*	46	22.4
Sleep disturbances	*0*	23	11.2
	*1*	168	82.0
	*2*	14	6.8
	*3*	0	0
Sleep latency	*0*	24	11.7
	*1*	107	52.2
	*2*	50	24.4
	*3*	24	11.7
Daytime dysfunction	*0*	49	23.9
	*1*	144	70.2
	*2*	10	4.9
	*3*	2	1.0
Sleep efficiency	*> 85%*	85	41.5
	*75–84%*	71	34.6
	*65–74%*	33	16.1
	*< 65%*	16	7.8
Sleep quality	*Very good*	7	3.4
	*Fairly good*	134	65.4
	*Fairly bad*	57	27.8
	*Very bad*	7	3.4
Sleep medication	*Not during the past month*	202	98.5
	*Less than once a week*	1	0.5
	*Once or twice a week*	1	0.5
	*Three or more times a week*	1	0.5
Overall PSQI median score [IQR]		6.0 [5.0–9.0]	

The intensity of pruritus was significantly associated with poorer sleep quality (Spearman’s rho = 0.408, *p* = < 0.001). Patients with moderate to severe pruritus were found to be 5.47 times more likely to exhibit bad sleep as compared to those with mild or no pruritus. No significant association was found between demographic and clinical characteristics of patients with the severity of pruritus.

## Discussion

The prevalence of CKD-aP was high, of which the majority of patients reported that it was mild in severity. The intensity of pruritus was significantly associated with poorer sleep quality.

The prevalence of CKD-aP in our study was 61.3%, which was similar to previous studies in Malaysia which reported rates of 58.6% [27] and 64.2% [28]; Turkey (53.4 and 85.4%) [29, 30] and74% in Pakistan [31]. The possible reasons for the variation in the prevalence of CKD-aP among different studies may be due to the study design, cohort studied (sample size, different study population), ethnicity (i.e. the individual’s tolerance or threshold for itch) or instruments used to assess CKD-aP [4, 32]. Moreover, the assessment of pruritus is also subjective one as it is based on perception, which is influenced by cultural, educational and socio-economic status [32]. Patients with depressive symptoms were reported to have 1.3 to 1.7 times higher risk of developing severe pruritus compared to those without depression [15, 33]. Patients with increased pruritus severity were also found to be more likely to miss hemodialysis sessions. Missed hemodialysis sessions have been associated with increased all-cause, cardiac-related, and infection-related hospitalizations and mortality [34].

The prevalence of CKD-aP in our study was 61.3%. However, the majority (86.3%) reported that their pruritus was mild. Our findings differed from previous studies which reported prevalence rates of mild pruritus as 48.8% in Japan [35], 22.2% in Egypt [36], and 41.7% in Saudi Arabia [37]. In our setting, only high-flux dialyzers were used, and studies have shown that high-flux dialyzers efficiently remove average-sized molecules associated with the aggravation of pruritus better than low-flux dialyzers [38]. However, some studies reported a high prevalence of CKD-aP even when patients were on high flux dialyzers: 62.6% in Taiwan [39], 53.4% in Turkey [30], 72.9% in Japan [38], 74.3% in Israel [40]. This may be due to the different type of dialyzer membranes used. Patients on dialyzers which uses polysulphone membranes experienced more pruritus than those using haemophane or cuprophane membranes [13, 41]. Some pruritogenic substances may be activated or released in greater amounts after blood contact with polysulphone membranes compared to other materials [42]. Use of high-flux polyacrylonitrile membrane has been reported to alleviate the severity of pruritus [43]. Hemodialysis with the target of Kt/V ≥ 1.5 (K = dialyzer clearance of urea, t = dialysis time, V = volume of distribution of urea, approximately equal to patient’s total body water) [44], as well as the use of high-flux dialyzer, may play a role in reducing the severity of uremic pruritus [44–46]. A search of published literature found that other factors that were found to be associated with CKD-aP were elevated blood urea nitrogen (BUN) [38, 47, 48]; crystal deposition of calcium and phosphate on skin (due to hypercalcemia and hyperphosphatemia) [49]; high aluminum level [50]; secondary hyperparathyroidism [47]; erythropoietin deficiency, high ferritin level, lower transferrin [30] and xerosis [51]. However, we did not found any associations in our study.

In our study, 48.8% of patients reported occasional delays in falling asleep, followed by 9.8% with frequent delays in falling asleep affected due to CKD-aP. Our findings were similar with other studies, where more than 45% [15], 59.1% [52] and 70% [38] of patients suffering from moderate to severe CKD-aP were observed to have poor sleep quality. Patients with moderate to severe pruritus were found to be 5.47 times more likely to exhibit bad sleep as compared to patients with mild pruritus, which was lower than a previous study which reported an odds ratio of 8.4 times for poor sleep [1]. A possible reason may be due to the fact that the majority of our patients reported having mild pruritus as opposed to having moderate or severe pruritus [53].

A limitation of our study was that the data collected was not generalizable, as it was only collected from dialysis centers based in Kuala Lumpur. We also did not have an (age and sex-matched) control group for our population. However, the strength of our study was that we had a very good response rate.

## Conclusion

In Malaysia, the prevalence of CKD-aP was 61.3%, of which the pruritus experienced was mild. Patients with moderate to severe pruritus were found to be 5.47 times more likely to experience poor sleep quality as compared to patients with mild or no pruritus.

## Abbreviations

5D-IS: 5D itching scale
CI: Confidence interval
CKD: Chronic kidney disease
MUHREC: Monash University Human Research and Ethics Committee
PSQI: Pittsburgh sleep quality index
